# Results of medical treatment with psyllium, lactobacillus, and tryptophan (Plurilac® Trio) in obstructive defecation syndrome

**DOI:** 10.3389/fsurg.2024.1361049

**Published:** 2024-04-08

**Authors:** Sayali Valiyeva, Domenico Tiso, Paolo Cerri, Antonio Pisciaroli, Renato Pietroletti

**Affiliations:** ^1^ Department of Applied Clinical and Biotechnological Sciences, University of L’Aquila, L'Aquila, Italy; ^2^ Clinical Nutrition, “Villa Maria” Hospital, Rimini, Italy; ^3^ General Surgery Department, Val Vibrata Hospital, Sant’Omero, Italy

**Keywords:** obstructive defecation syndrome, defecation disorder, chronic constipation, psyllium fiber, quality of life

## Abstract

**Introduction:**

The term “obstructive defecation syndrome” (ODS) describes a complex condition characterized by defecatory disorders. Such a condition represents a significant proportion of patients, which is estimated to be up to 30% of patients affected by chronic constipation. Presently, a broad agreement has been reached on diagnostic studies, whereas the choice of treatment that aims to improve the quality of life and/or correct the prevalent abnormalities or all anatomical abnormalities remains controversial.

**Methods:**

This was a retrospective cohort study on 174 patients out of a total of 232 with ODS who were observed in a specialized university unit of surgical coloproctology between 2018 and 2022. Clinical assessment included examining the values of the Agachan–Wexner constipation score and Patient Assessment of Constipation (PAC)-quality of life (QoL) scores, a full digital anorectal examination, anoscopy, RX defecography, and a urogynecological consultation; a functional anorectal test, an endoanal ultrasound, and colonoscopy were performed in select patients. The patients were reevaluated after an 8–10-week course of medical treatment based on a high-fiber diet and fluid intake and 6 g of psyllium combined with lactobacillus and tryptophan b.i.d. The results were analyzed by means of the Wilcoxon rank-sum test, comparing pretreatment scores with the results at the first follow-up visit.

**Results:**

After 8–10 weeks of conservative treatment, 128 patients declared full satisfaction, 29 reported moderate satisfaction, and 17 (9.7%) declared no improvement. Among these 17, there were 5 patients with paradoxical puborectal contractions. The value of the Agachan–Wexner constipation score after treatment decreased from the pretreatment Agachan–Wexner constipation score mean value of 23.4 ± 3.7 (mean ± SD range 15–27) to a mean value of 5.3 ± 0.7 (range 3–8, *p* < 0.001). The quality of life improved, as shown by the PAC-QoL score, indicating great improvement in social relationships.

**Conclusions:**

Given the benefits of conservative therapies, they represent a cornerstone in the treatment of ODS, a complex disorder. Diet and bulking agents are mandatory forms of treatment prior to making any surgical attempt, also considering the fact that the psychosomatic component of ODS is an essential prerequisite to match patient expectations.

## Introduction

The term “obstructive defecation syndrome” (ODS) describes a complex condition characterized by defecatory disorders. Such a condition represents a significant proportion of patients, which is estimated to be up to 30% (1, 2) of patients affected by chronic constipation.

Symptoms of straining at defecation, incomplete evacuations, frequent daily toilet visits, self-manipulation, and perineal discomfort are prevalent in these patients and are associated with those of “functional constipation,” as depicted in “Rome IV criteria” (3). Such patients often show features of psychological discomfort, mainly because of the impact of ODS on life and social relationships (4). However, a conflictual personality and stressful events in life may be determinant triggers of functional bowel disorders and/or disturbed defecatory behavior (5–7). For these patients, a multidisciplinary study that investigates the dual interaction between the psyche and the intestine and how this affects their lives may be suggested (8).

Anorectal functional investigations and/or imaging studies commonly show abnormal findings in these patients, often secondary to long-standing symptoms of ODS such as chronic straining and prolonged defecation time. Surgical treatments that aim to correct such abnormalities may contribute to alleviating ODS and thus improve the quality of life (QoL) (9).

Presently, widespread agreement has been reached on diagnostic studies and the adoption of a conservative approach to the treatment of ODS that includes diet, laxatives, and rehabilitation.

Conversely, the choice of the most appropriate and personalized treatment that aims to correct the prevalent abnormalities or all anatomical abnormalities remains controversial (10).

In our studies, we report a retrospective analysis of patients with ODS, drawing attention to the results of a first-line conservative protocol.

## Materials and methods

During the period between 2018 and 2022, we observed, in a specialized university unit of surgical coloproctology, 232 patients who complained of ODS. Of these, 58 showed significant anatomical abnormalities on imaging studies and did not respond to conservative treatment, following which surgical treatment was indicated. Therefore, they were not included in the study, which is based on the analysis of patients who responded to conservative measures using a specific bulking laxative (Plurilac Trio).

The remaining 174 patients represent the case series considered in the present investigation. In this cohort, there were 164 women and 10 men with a mean age of 58.4 years (range 18–79). Of these 164, increased BMI was observed in 125 (76.2%). Of these, 91 were in the overweight range, and the remaining 34 were in level 1 obesity (72.8% and 27.2%, respectively). Only 3 out of 10 male patients showed an increased BMI in the range of 25–29.9. The clinical features of this patient series are summarized in Table 1.

**Table 1:** The clinical features of the patients.

Total No.	174 (164 Female/10 Male patients)
Mean age	58.4 (18–79)
Prolonged defecation time (20–40 min)	100%
Feeling of incomplete evacuation	95%
Repeated daily toilet visits	54%
Excessive straining (>50% of the time)	87%
Self-digitation	32%
Laxative abuse/enema	76%/12%
Rectocele	41%
Rectocele + intussusception	54%
Intussusception alone (10 males patients)	5.8%

Clinical assessment of all patients included a full digital anorectal examination (basal sphincter tone, squeeze, and straining), anoscopy, RX defecography, and a urogynecological consultation when indicated. A functional anorectal test and an endoanal ultrasound were performed in those with a history of trauma at delivery and/or anal surgery, 68% of patients in total. A colonoscopy was performed if indicated by age older than 50 years and/or familial history in a total of 73% of patients.

The evaluation of ODS was based on the Agachan–Wexner score (11) and defecography. The impact of disturbed defecation on the patients’ quality of life was assessed by means of a Patient Assessment of Constipation (PAC)-QoL questionnaire. This scoring system is in the form of a validated questionnaire comprising 28 questions with multiple answers and four subscales (worries and concerns, physical discomfort, psychosocial discomfort, and satisfaction), which is reproducible and adopted globally, with scores ranging from 0 to 112 (12).

The basic treatment included a high-fiber diet, adequate liquid intake, and a bulking agent containing psyllium, tryptophan, and *Lactobacillus acidophilus*. Patients on chronic treatment with pharmacological laxatives or enema were encouraged to withdraw progressively according to the following plan: intake of laxatives every other day for 1 week, every 2 days for 1 week, once a week for 2 weeks, and then stop intake. Rehabilitation was added to this protocol in 12 patients (9 men) who had paradoxical puborectal contractions.

The patients were reevaluated after an 8–10-week course of medical treatment by means of the Agachan–Wexner constipation score and the PAC-QoL questionnaire.

### Statistical analysis

The results were analyzed by means of the Wilcoxon rank-sum test, comparing pretreatment scores with the results at the first follow-up visit. Data are reported as the mean and standard deviation of the mean. A *p*-value <0.05 was considered statistically significant.

## Results

The clinical symptoms of ODS reportedly varied in all patients. To elaborate, straining at defecation (more than 50% of the time) and prolonged defecation time (sitting for more than 20 min in the toilet) were declared by 87% and 100% of patients, respectively. A total of 43% reported frequent and daily toilet visits, and 32% complained of self-digitation. The clinical features of the patient series are summarized in Table 1.

A defecating proctogram showed the presence of a rectocele, coupled with rectal intussusception, in 54% of the patients. Rectocele alone was reported in 41% of the patients. The 10 male patients showed intussusception alone.

The pretreatment Agachan–Wexner constipation score showed a mean value of 23.4 ± 3.7 (mean ± SD, range 15–27).

The PAC-QoL score indicated a negative impact of the defecatory disorder on social and working life. The use/abuse of laxatives was reported by 76% of patients, whereas 12% adopted enemas ranging from daily use to every other day. After 8–10 weeks of conservative treatment, 128 (73.6%) patients declared full satisfaction with treatment, 29 (16.6%) reported moderate satisfaction, and 17 (9.7%) declared no improvement. Among the non responders, 9 were affected by paradoxical puborectal contractions out of 12. Gradual weaning from laxatives in this patients proved unsuccessful, following which all of them reprised the intake of laxatives. The value of the Agachan–Wexner constipation score after treatment was reduced to a mean value of 5.3 ± 0.7 (range 3–8, *p* < 0.001). No correlation was found between response to treatment and BMI.

**Figure 1:**
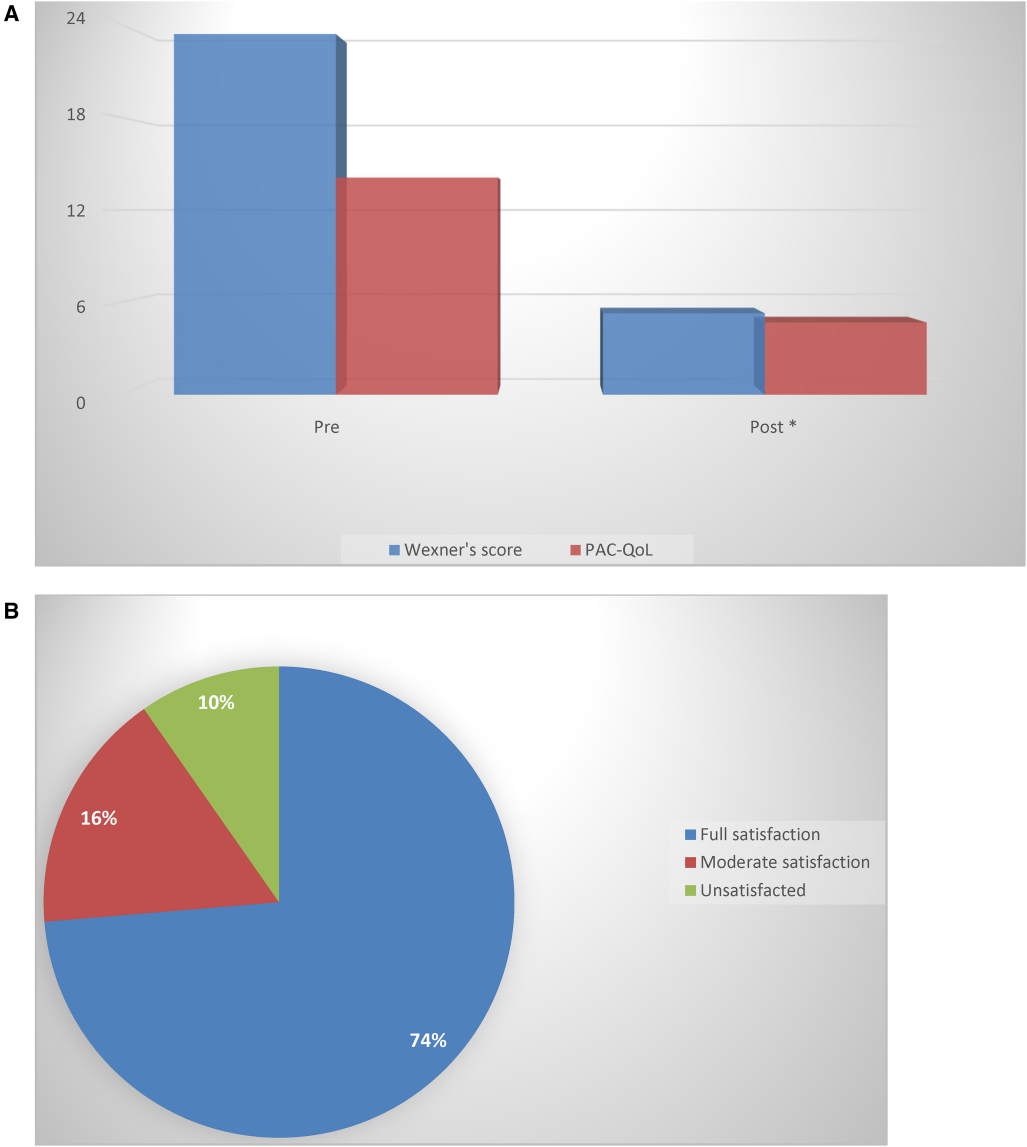
(**A**) A comparison of the Agachan-Wexner score and PAC-QoL questionnaire before and after treatment (*p < 0.05) (Wilcoxon rank-sum test). (**B**) The percentage of patients declaring satisfaction or dissatisfaction with the conservative treatment adopted.

Improvement in the quality of life, as shown by the PAC-QoL score, indicated great improvement in social relationships. The results are summarized in Figure 1.

## Discussion

ODS is a complex functional syndrome frequently encountered in women, deeply affecting the quality of life of patients. Its pathophysiology has not been fully elucidated because different abnormalities, of both functional and/or anatomical origins, have been observed, all concurring in different degrees with the clinical manifestation, and therefore, the treatment of ODS is challenging.

High resting anal pressure (13, 14), rectal hyposensitivity (15), or anatomical defects such as rectocele, intussusception, and perineal descent (16, 17) are factors considered responsible for the occurrence of ODS. The factors of female sex and obesity (94.3% and 73.5%, respectively, in the whole series) predominate for the obvious, associated reasons of pelvic floor distress. However, to what extent such sensory–motor–anatomical defects may cause defecatory disturbances remains unclear. It has been hypothesized that they may represent merely a consequence of ODS rather than the cause itself (18).

However, regardless of the controversy existing in the diagnosis and treatment of ODS, medical management with the adoption of a diet plan and with bulking and/or osmotic laxatives can lead to a significant improvement in this condition. In fact, we observed an improvement in symptoms in more than 90% of our patients, with only a small percentage of them being dissatisfied. Interestingly, in this group of dissatisfied patients, approximately 50% of them showed features of paradoxical puborectal contraction. Regadas et al. (19) showed how biofeedback, combined with diet, could be a valuable treatment option for patients with ODS associated with anismus.

Such patients need rehabilitation plans devised by skilled professionals, but regrettably, we must admit that such expertise is not widely available as yet, or conversely, rehabilitation plans are not implemented properly. In this context, Barucha et al. (10, 17) caution against the implementation of an inappropriate “sphincter strengthening exercise” instead of coordination activities to treat patients with ODS.

The water-soluble dietary fibers adopted in our treatment protocol, in accordance with recent evidence (20), showed excellent results. The guidelines suggest fiber supplementation with 25–30 g per day. A 4-week plan of soluble dietary fiber supplements accelerates the colon transit time and alleviates clinical symptoms in patients with slow-transit constipation (21). In addition, supplementary fiber offers protective effects on the gut microbiota by increasing the population of healthy microflora (20). However, the use of stimulant laxatives other than soluble fibers or osmotic agents should not be stopped even in the long term. Bisacodyl, senna, and pico-sulfate exert positive effects on colonic motility, counteracting the negative effect of delayed or incomplete evacuation (22–27).

Such substances in long-term experimental studies on rats were not found to cause any damage to the enteric nerve plexus. Therefore, they may be employed on a regular basis or occasionally as a rescue treatment after 2–4 days of a patient experiencing difficulties (28, 29).

We adopted a treatment protocol of 8–10 weeks, which is double the time span adopted in the majority of the trials with laxative use because we discouraged the constant use of pharmacologic laxatives. In fact, apart from the evidence of no harm to enteric nerve plexuses, the receptor interaction of such substances may produce an upregulation phenomenon, thus reducing long-term effects (30).

Recent studies have demonstrated a causal relationship among constipation, dysbiosis, and intestinal peristalsis. Cao et al. (31) suggest that gut dysbiosis could inhibit intestinal motility and contribute to the development and persistence of constipation. The authors provide a point of view to demonstrate the pathogenesis of constipation, in addition to hypothesizing the need for innovative microbiota-mediated therapy for treating chronic constipation (31).

According to the most recent literature, patients suffering from constipation should be treated with a multitarget therapy that acts on both the volume and the softness of the fecal mass (soluble fiber) and on gut motility and microbiota (32).

The product employed in our protocol is a combination of psyllium, tryptophan, and *Lactobacillus acidophilus*, which has a wide range of effects aimed at restoring colonic function.

Psyllium is a widely recognized water-soluble fiber that acts as a bulking agent and stool softener and improves defecation. Doses above 10 g/day and a treatment duration of at least 4 weeks appear optimal (33). Clinical data on psyllium show that it has excellent tolerability, especially with regard to side effects. Some of these effects (bloating, flatulence, etc.) are particularly disturbing, but their rate of incidence is not significantly different from that of placebo (34).

Psyllium, being moderately fermentable, has a prebiotic action on the resident microbiota. As a result, the gut microbiota lowers the luminal pH and produces a series of bioproducts, the most important of which is a short-chain fatty acid called “butyrate,” which influences the neuroendocrine system by promoting gastrointestinal secretion and motility (35).

There is increasing evidence of a causal relationship between constipation and dysbiosis (36, 37). The presence of Lactobacillus acidophilus in the compound helps to restore the normal balance of intestinal bacteria in the colon.

There is no doubt that prolonged storage of feces in the intestine can alter the composition of the microbiota. Clinical studies have shown that the bacteroides, Clostridium difficile, and Bifidobacterium, are more abundant in the colon mucosa of patients with chronic constipation, at the expense of *Lactobacillus* and *Faecalibacterium* (31).

Scientific evidence shows that intestinal dysbiosis can contribute to the development of chronic constipation through modulation of the serotoninergic pathway (38).

It emerges, therefore, that there is not only a correlation between constipation and dysbiosis but also a correlation between dysbiosis and intestinal motility (32). In this respect, the combination of psyllium with *Lactobacillus acidophilus* and tryptophan may exert multiple effects as a regulator of gut motility and microbiota (31, 32, 38–40).

Tryptophan is an amino-acid precursor of amine and neurotransmitter syntheses, both of which are essential for the proper functioning of endocrine cells and nerve plexuses and for the tropism of colonocytes. Tryptophan is a precursor of the neurotransmitter serotonin (39). Serotonin is a biogenic amine synthesized mainly in the gastrointestinal tract that, once released in response to mechanical and chemical stimulation, activates receptors that are located on the presynaptic terminals of enteric nerve cells. The stimulation of serotonin receptors increases the release of peptides related to the acetylcholine gene (which causes smooth muscle contraction ) and calcitonin (which causes smooth muscle relaxation ) from the nerve terminals. This action strengthens neurotransmission in the prokinetic pathways, improving gastrointestinal motility (40).

The properties of the combination of psyllium, *Lactobacillus acidophilus*, and tryptophan exert a multifactorial effect, not only in terms of bowel normalization but also in terms of providing a significant alleviation of abdominal symptoms such as pain, bloating (different from psyllium alone), flatulence, and perineal discomfort, which are often reported by patients and that contribute to poor quality of life.

Rehabilitation of patients with obstructed defecation provides the opportunity to reduce the severity of symptoms in some patients. Chiarioni et al. demonstrated that biofeedback ameliorated symptoms and accelerated bowel transit in over 70% of patients with slow-transit constipation caused by dyssynergia, while patients with an isolated impairment of gut transit did not show any improvement (41). Pucciani et al. (42) showed a synergic effect of psyllium with rehabilitation, which improved rectal sensation compared with only a high-fiber diet plan. Success rates vary widely, but only limited data are available on the factors predictive of success. It is recommended that a psychiatric evaluation be carried out, especially before starting rehabilitation therapy for obstructed defecation, because the presence of psychiatric disorders could alter the course of, and decrease the efficacy of, such a rehabilitation program (43).

In conclusion, in the management of ODS, many challenges exist. Given the non-negligible benefits of conservative therapies, such therapies should be attempted prior to the surgical management of ODS, whether they are transanal, transvaginal, transperineal, or transabdominal in nature (44). As is known, surgical site infection is the most common postoperative complication of colorectal surgery, causing pain and suffering to patients in the form of negative economic impact, increased morbidity, extended postoperative hospital stay, readmission, sepsis, and death (45, 46). Therefore, conservative therapy helps us to be very selective. Because of the small size of the patient sample in this study, our conservative treatment protocol, which includes psyllium with tryptophan and lactobacillus acidophilus, yields good results in terms of alleviation of symptoms and improvement in quality of life.

However, many unsolved questions remain, such as the role of gastrointestinal hormones, the microbiota, or other psycho–neuro–endocrine–biological mechanisms in obstructed defecation. In this respect, therapeutic possibilities are being explored in the area of microbiota manipulation, including fecal transplants.

## Data Availability

The original contributions presented in the study are included in the article/Supplementary Material, and further inquiries can be directed to the corresponding author.
